# Use of plasmapheresis during cardiopulmonary bypass in a pediatric heart transplant of a patient with Failing Fontan Physiology: first case in Argentina

**DOI:** 10.1051/ject/2025053

**Published:** 2026-03-13

**Authors:** Matias Jorge Martinez, Ignacio Berra, Javier Cornelis, Juan Costilla, Fernando Zamora, Pablo Garcia Delucis

**Affiliations:** 1 Cardiovascular Surgery, Heart Transplant and Peripheral Vascular Service. Prof. Dr. Juan P. Garrahan Pediatric Hospital Combate de los Pozos 1881 (1245) CABA Buenos Aires Argentina; 2 Hemotherapy and Transfusion Medicine Service. Prof. Dr. Juan P. Garrahan Pediatric Hospital, Combate de los Pozos 1881 (1245) CABA Buenos Aires Argentina

**Keywords:** HLA, Antibodies, Heart transplant, Plasmapheresis, Cardiopulmonary bypass

## Abstract

*Background*: A 17-year-old male patient diagnosed with a single ventricle, in a failed Fontan stage, was evaluated prior to heart transplantation. The patient had a panel-reactive antibody (PRA) for human leucocyte antigen (HLA) I of 18% and for HLA II of 37%, so the decision was made to administer three doses of immunoglobulin while waiting for a donor heart. *Methods*: Once extracorporeal circulation was initiated, the apheresis machine extracted blood from the patient’s venous drainage and returned it to the oxygenator reservoir. A total of 8278 mL of blood was processed, and 4224 mL of plasma was extracted. For replacement, 1341 mL of fresh frozen plasma and 2700 mL of 5% albumin were used. 75 mL of citrate-dextrose acid (CDA) was used as an anticoagulant. The procedure lasted 135 min. *Results*: On the tenth postoperative day, the PRA for HLA I and II was 0%. On the thirtieth postoperative day, a catheterization with endomyocardial biopsy showed no evidence of immunological rejection. An echocardiogram showed good function of the heart graft. One year later, a catheterization with endomyocardial biopsy showed no signs of humoral rejection. The patient is currently in the third-year post-transplant and continues to show no signs of rejection in their progression. *Conclusion*: Plasmapheresis during cardiopulmonary bypass is a reproducible, safe, and effective technique. It may be indicated for sensitized patients on the heart transplant waiting list.

## Introduction

### HLA and therapeutic plasma exchange

The human leukocyte antigens (HLA) system includes a complex variety of genes located within the Major histocompatibility complex (MHC) on the short arm of chromosome 6, along with their molecular products, which are involved in immune regulation and cellular differentiation. HLA molecules are expressed on nearly all nucleated cells and are the primary molecules that initiate graft rejection. HLA antigens are divided into two groups (Class I and Class II). The HLA-A, HLA-B, and HLA-C genes encode the corresponding Class I antigens A, B, and C. The HLA-DR, HLA-DQ, and HLA-DP genetic groups encode the synthesis of the Class II antigens with the same names. Class I molecules are found on the surface of platelets and most nucleated cells in the body. Class II antigens are limited to a few cell types, such as B lymphocytes, monocytes, macrophages, dendritic cells, and activated T lymphocytes [1].

When a human transplant is performed, the HLA molecules of a donor are recognized by the immune system of the recipient through direct and indirect allo-recognition methods that trigger an alloimmune response. The compatibility between the donor and recipient for MHC antigens has a significant positive effect on graft acceptance. In organ transplantation, adaptive immunity is the primary response to the transplanted tissue, as the main target of the immune response is the MHC molecules expressed on the surface of the donor’s cells. Activation of T cells leads to the production of cytokines and chemokines, which in turn can recruit components of innate immunity such as NK cells, macrophages, and the complement system. Additionally, defensins and cathelicidins have chemotactic properties on T lymphocytes.

In transplant immunology, the greatest impact on graft loss comes from the effects of HLA-B and -DR antigens. The effects of HLA-DR mismatches are most significant in the first 6 months after transplantation, the HLA-B effect arises in the first 2 years, and HLA-A mismatches have a detrimental effect on long-term graft survival [2].

HLA molecules constitute one of the two major immunological barriers (the other being ABO antigens) to be analyzed prior to a solid organ transplant. The presence of these antibodies in the recipient’s serum is responsible for hyperacute rejections [1].

Regarding congenital heart disease and HLA antibodies, an elevated PRA has been reported to occur in 12% to 19% pediatric patients transplanted for end-stage congenital heart disease. The proportion of children transplanted with congenital heart disease is highest in the infant population (63%) but remains substantial in the adolescent age group (24%). These patients have often undergone multiple prior congenital heart surgeries and received blood transfusions, predisposing them to antibody formation. Shaddy et al. [3] and Hawkins et al. [4] characterized the formation of HLA class I and class II antibodies after implantation of cryopreserved allograft material for the repair or palliation of congenital heart disease. Within 3 months of implantation, the mean class I and II antibody levels reached 92 ± 15% and 70 ± 26%, respectively, and the high levels of circulating antibodies persisted for at least 12 months. The presence of allosensitizing material likely contributes to the higher incidence of HLA antibodies detected in children with congenital heart disease [5].

Current strategies to decrease allosensitization focus on the direct removal of circulating antibodies with plasmapheresis, inactivation of antibodies with IVIG, and B-lymphocyte depletion with rituximab. These approaches have also been used to treat antibody-mediated rejection after transplantation with encouraging results [6].

Plasma of the patient is separated from other components of blood, either by membrane filtration (mTPE) or centrifugation (cTPE). The plasma is removed with subsequent substitution of a replacement solution (e.g., human albumin and/or plasma) or a combination of crystalloid/colloid solution. In the literature, plasmapheresis is often used synonymously with TPE. The term high-volume TPE (TPE-HV) is used if > 2 plasma volumes are exchanged in a single session [7].

**Figure 1:**
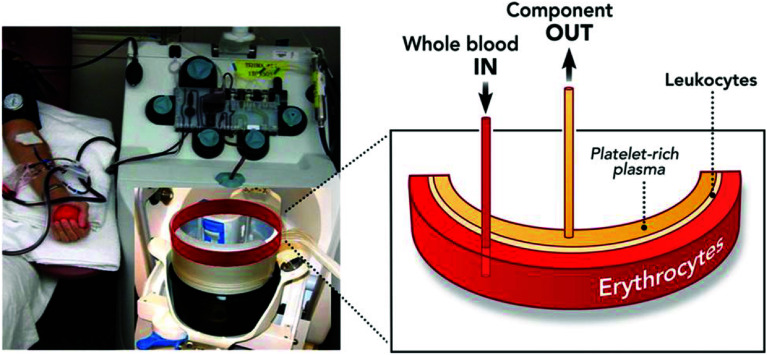
Separation of blood components in the apheresis machine.

The plasma exchange procedure is performed using a continuous-flow cell separator, which is connected to the patient via two lines (extraction and return) through a dual-lumen central venous access. In particular situations, the connection may be made to another extracorporeal circulation device that is connected to the patient (such as a hemofilter, cardiopulmonary bypass machine, etc.). Blood is extracted from the patient through one line while the cellular components are mixed with the replacement fluid and returned to the patient through the other line (Figure 1).

The amount of plasma to be exchanged is calculated based on the patient’s plasma volume, which is the difference between the total blood volume and the red cell mass. Once the total blood volume is obtained, and knowing the hematocrit value, the red cell mass can be calculated using the following formula:

Vol × Hematocrit /100 = Plasma volume.

The replacement fluid to be used depends on the patient’s condition. It is usually 5% human albumin. In cases of coagulopathy or prior to surgery, a combination of albumin and fresh frozen plasma is used.

Sodium citrate, the most commonly used anticoagulant in therapeutic apheresis, works by chelating ionized calcium and preventing clot formation. Current automated apheresis machines control the citrate infusion rate to achieve anticoagulation while minimizing the risk of hypocalcemia.

Therapeutic plasma exchange (TPE) may be indicated for:

- Patients with heparin-induced thrombocytopenia (HIT) with or without thrombosis (HITT).
- Patientswithantiphospholipidsíndrome.
- Hypersensitized patients undergoing heart transplantation [8].

Antibody-mediated rejection (AMR) after heart transplantation occurs in approximately 7–18% of recipients and is associated with acute cardiac graft dysfunction and increased risk of allograft failure. The major barrier to transplantation is anti-HLA antibodies, which are associated with AMR and post-transplant morbidity and mortality.

Current strategies to reduce the prevalence of posttransplant AMR in sensitized patients target the reduction of circulating donor-specific anti-HLA. During the immediate postoperative period, pre-sensitized heart transplant recipients may present with hemodynamic instability and hypoxemia because of AMR that requires extracorporeal membrane oxygenation (ECMO) support. Therefore, automated TPE in parallel with ECMO would be useful in this situation.

Susceptibility to infection from reduced immunoglobulin levels associated with TPE in AMR patients has not been clearly established. Jhang et al. [9] found that TPE in parallel with ECMO is technically possible, safe, and effective in reducing anti-HLA antibodies for the treatment of AMR of the transplanted heart. Another example of antibody clearance for possible rejection was demonstrated by Dellgren et al. [10] They showed that ABO-incompatible transplantation can be performed without significantly increased mortality or morbidity. There is an option of pre-emptive removal of anti-A and anti-B antibodies during plasma exchange on bypass before graft reperfusion and/or the low pre-transplantation antibody titers typical of recipients in the first year of life. Patients receiving ABO incompatible grafts showed a tendency to produce lower levels of antibodies directed against the incompatible blood group antigen than against the non-expressed antigen. In this group of patients, little or no evidence of vascular rejection was seen, and persistent cellular rejection did not occur during follow-up in recipients of ABO-incompatible grafts. An anecdotal example was reported by Adachi et al., who successfully conducted TPE via VAD circuitry in two pediatric patients who experienced humoral rejection following heart transplantation.

Additional studies are needed to determine the optimal timing, number, and frequency of TPE procedures, and to better establish a direct correlation between reduction in anti-HLA antibody titers and ventricular function and long-term outcome. However, given the high mortality associated with post-transplant AMR, especially in pediatric heart transplant recipients, early recognition and rapid treatment of acute post-transplant cardiac dysfunction should strongly be considered [11].

### Fontan failure

Patients with a single ventricle have reduced and less efficient transpulmonary blood flow compared to a biventricular circulation, especially in response to increased demands such as exercise. The chronic rise in systemic venous pressure and low cardiac output are responsible for end-organ dysfunction and even permanent damage. Therefore, it is very likely that almost all patients with Fontan physiology will develop serious adverse effects of this physiology (Fontan failure), such as valve insufficiency, residual aortic obstruction, arrhythmia, coronary insufficiency, or the development of aortopulmonary collaterals. On the other hand, there may be protein-losing enteropathy, plastic bronchitis, and cyanosis. Regardless of the dominant pathophysiology, Fontan physiology can lead to liver dysfunction and cirrhosis, esophageal varices, renal dysfunction, nutritional disorders, and thromboembolic events. Some patients with poor Fontan physiology may respond to medical therapy, while in some patients with severe end-organ dysfunction, the use of mechanical circulatory support may reverse the dysfunction and improve the patient’s condition to allow them to survive until a heart transplant [12, 13].

## Materials and methods

Our case involved a 17-year-old male patient who weighed 57 kg and was 160 cm tall. He was diagnosed with ambiguous atrial situs, L-loop ventricular, L-position of the aorta, levocardia, total anomalous pulmonary venous return (TAPVR) with tricuspid atresia, transposed vessels with pulmonary atresia (IIA), heterotaxy syndrome with dextroisomerism, and levocardia. The patient had severe insufficiency in the AV valve and a dilated single ventricle with severe ventricular dysfunction. The extracardiac tube contained a permeable stent inside measuring 12 × 18 mm. The double Glenn procedure was performed (the right side was smaller with lower velocity).

**Figure 2:**
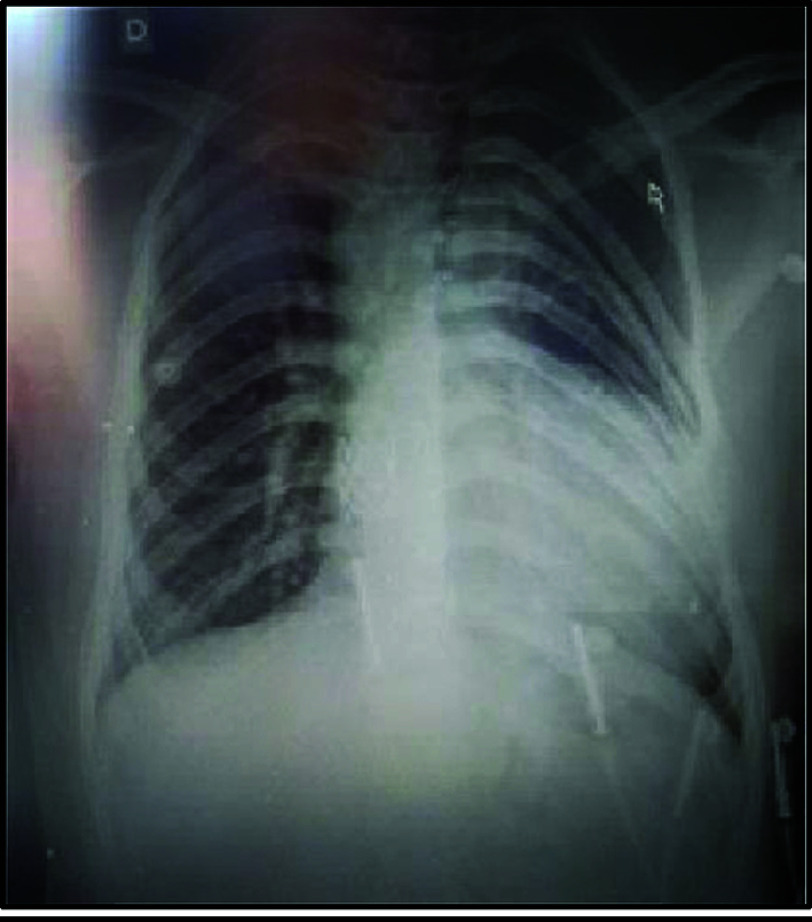
Frontal chest X-ray.

At one year of age, the patient underwent bilateral systemic-pulmonary anastomosis. At three years, a fenestrated extracardiac conduit was placed, which corrected the total anomalous pulmonary venous return, along with pulmonary branch plastic surgery and bilateral Glenn surgery. At eight years, the fenestration of the extracardiac conduit was closed. At 17 years, the patient was placed on the heart transplant list due to dysfunctional Fontan physiology with severe AV valve insufficiency and moderate to severe dysfunction of the single ventricle. In the chest X-ray (Figure 2), cardiomegaly and the stent of the extracardiac tube were evident (Figure 1).

When the patient was on the transplant list, we planned plasmapheresis during cardiopulmonary bypass and conducted a simulation in the operating room to assess the anticoagulation of both circuits, the apheresis connections with the CPB, and the management of total volumes.

For the perfusion, the Stockert S5 heart-lung machine and a Stockert 3T heat exchanger (LivaNova PLC, 20 Eastbourne Terrace, London, W2 6LG, United Kingdom) were used. A LivaNova Inspire 6F oxygenator, Sorin tubing with both venous and arterial lines (3/8″), and a hemoconcentrator DHF 02 (LivaNova PLC, 20 Eastbourne Terrace, London, W2 6LG, United Kingdom) were employed.

The priming of the circuit consisted of 1000 mL of neutral pH, isosmolar polyelectrolyte solution (Rivero Laboratories, Avenida Boyacá 419 (C1406BGH) Buenos Aires, Argentina), 200 mL of 20% albumin, 100 mL of 1 M sodium bicarbonate, 1 g of cefalotin, and 10,000 IU of sodium heparin.

Due to the patient’s elevated hematocrit (56%), an acute normovolemic hemodilution of 500 mL was performed prior to the start of the perfusion.

For the TPE, a Spectra Optia cellular separator was used, along with a plasmapheresis circuit with a total volume of 236 mL.

**Figure 3:**
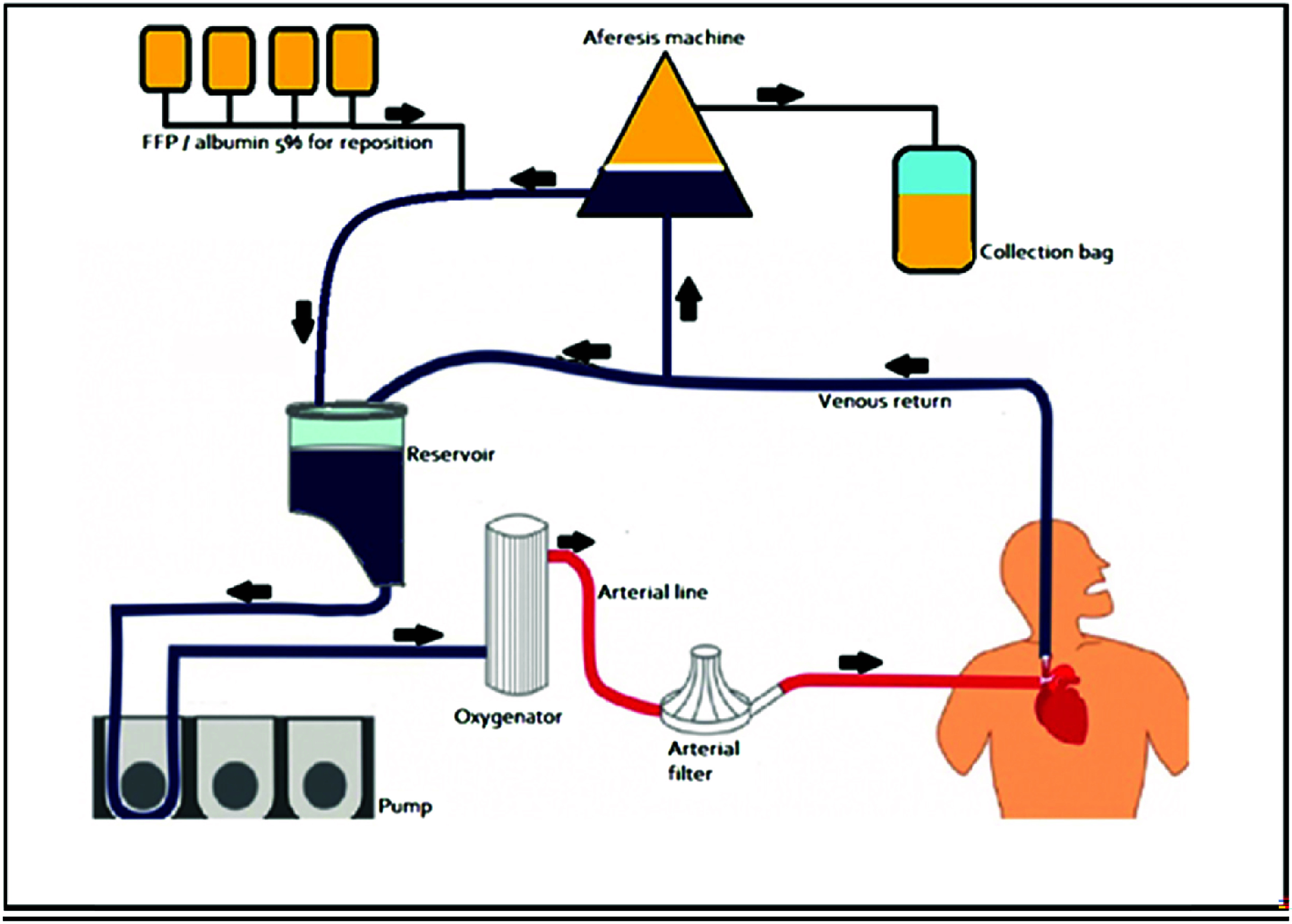
Diagram of the apheresis circuit connected to the CPB.

In the section of the tubing of the patient’s venous drainage closest to the reservoir, a connector with a luer and a three-way topcock was placed to allow the blood to be diverted to the apheresis machine. After the plasma was processed and discarded, the blood, along with the replacement fluids, returned to the extracorporeal circuit through a luer connection on the reservoir, with or without a three-way topcock (Figure 3). It was important to note that the apheresis machine was primed before starting operation.

During the sternotomy, bleeding occurred due to adhesions of the heart to the bone. It was decided to initiate cardiopulmonary bypass (CPB) via the femoral artery and vein and to cool the body to 22 °C to preserve the vital organs. After controlling the bleeding, TPE was started.

The patient’s plasma volume was 2074 mL. It was decided to exchange this volume twice. The replacement with 1341 mL of fresh frozen plasma at a flow rate of 30 mL/min lasted 75 min. Subsequently, the replacement with 2700 mL of 5% albumin took 60 min at a flow rate of 100 mL/min. A total of 75 mL of CDA was used as an anticoagulant. The total volume of blood processed was 8278 mL, with 4224 mL of plasma extracted. The procedure lasted 135 min and was completed before the aortic clamp was removed.

Once CPB was initiated, femoral venous cannulation and venous drainage allowed an initial flow of 2 L/min with the heart beating and the lungs ventilated. After completing the sternotomy and controlling the bleeding, central cannulation of the vessels was performed, and the temperature and pump flow were gradually increased to 32 °C and 4.0 L/min, respectively. The patient’s mean arterial pressure was 60 mmHg.

During CPB, six laboratory samples were taken – three arterial and three venous – maintaining a hematocrit of 27%, a venous oxygen saturation of 83%, and an average lactate level of 2 mmol/L.

Regarding anticoagulation management, the amount of heparin used in the priming was in accordance with the daily protocol. When the patient’s temperature reached 32 °C, 15.000 IU of heparin were added to achieve an activated clotting time (ACT) greater than 600 s. The ACT was measured according to the usual protocol, as no adverse events related to routine anticoagulation were observed during the previously mentioned simulation.

The calcium level, which was significantly low during CPB and TPE (0.6 mmol/L) due to the CDA used in the TPE, was corrected to normal values once the patient reached the range of mild hypothermia (35 °C), prior to the termination of extracorporeal circulation.

The patient did not receive any transfusions during CPB. The ultrafiltration volume was 2000 mL. Urine output was 500 mL.

The cell saver was used from the skin incision until the initiation of cardiopulmonary bypass. After the administration of protamine, it was used again until the placement of the sternum mesh. The entire residual volume of the extracorporeal circuit was processed. Total volume processed was 2000 mL.

## Results

In the immediate postoperative period, high doses of inotropes and vasopressors were administered. On physical examination, cold extremities, slow capillary refill, and low urine output were observed. The patient also required transfusions of red blood cells, fresh frozen plasma (added to that used in TPE), and platelets. All components were irradiated and leukocyte depleted.

During the first eleven days postoperatively (in the ICU), four TPEs were performed without complications. Good ventricular function, improvement in renal function, and withdrawal of oxygen and inotropes were observed.

In the four days following discharge from the ICU, the final TPE was performed, and two doses of 50 grams of immunoglobulin were administered.

On the tenth postoperative day, the first postoperative PRA for HLA I and II was 0%. On the thirtieth postoperative day, a catheterization with endomyocardial biopsy showed no evidence of immunological rejection. An echocardiogram showed good graft function. One year later, a catheterization with endomyocardial biopsy showed no signs of humoral rejection. At the time of publication of this article, the patient is in the third-year post-transplant and continues to show no signs of rejection in his progress.

## Discussion

Organ transplants have helped thousands of people. The main obstacle lies in the availability of organs, so it is necessary to increase the number of available organs and to find more precise immunosuppressive methods to prevent rejection without the dangerous side effects of infections and cancer.

The role of HLA compatibility in transplant outcomes is considered [2].

Sensitization remains a limitation for heart transplantation in many potential candidates. Data on current desensitization therapies and their outcomes are limited, despite the strong rationale. Future research should focus on understanding the outcomes of desensitization therapy and evaluating both current and new therapies [3].

The combination of plasmapheresis and cardiopulmonary bypass allows for the removal of cytotoxic antibodies in patients with positive PRA. The therapeutic effect was sustained when combined with aggressive B-cell immunosuppressive therapy. Plasmapheresis during CPB provided high-flow exchange, which would not be possible in these hemodynamically unstable patients. A major issue when performing plasmapheresis during CPB is the removal of various drugs, such as heparin, anesthetics, aprotinin, steroids, and ionized calcium. In summary, plasmapheresis during CPB offers easy access to cardiopulmonary bypass connections and stable volume management but requires special attention to the serum factors removed during plasmapheresis.

The frequency and volume of post-transplant plasmapheresis should be guided by the PRA results following each plasmapheresis procedure. Without aggressive post-transplant plasmapheresis and B-cell-specific immunosuppressive therapy, this procedure may have only a short-term effect due to a possible B-cell response to the allograft [14].

## Conclusion

Therapeutic plasma exchange during extracorporeal circulation can serve as an adjunct to immunosuppression in the treatment or prevention of antibody-mediated rejection in solid organ transplants. TPE is a reproducible, safe, and effective technique. It may be indicated for sensitized patients on the heart transplant waiting list and, in combination with immunomodulatory therapies such as intravenous immunoglobulin before a transplant, can reduce the risk of rejection in HLA-immunized patients. This therapy was successfully performed for the first time in Argentina, in a public hospital, on a patient with congenital heart disease and a failed Fontan, with positive results.

## Data Availability

All available data are included in the article.
